# Branched-chain amino acids: physico-chemical properties, industrial synthesis and role in signaling, metabolism and energy production

**DOI:** 10.1007/s00726-024-03417-2

**Published:** 2024-08-28

**Authors:** Philipp Reifenberg, Aline Zimmer

**Affiliations:** 1 Merck Life Science KGaA, Upstream R&D, Frankfurter Strasse 250, 64293 Darmstadt, Germany; 2 Institute for Organic Chemistry and Biochemistry, Technische Universität Darmstadt, Alarich‑Weiss‑Strasse 4, 64287 Darmstadt, Germany

**Keywords:** Branched-chain amino acids, Isoleucine, Leucine, Valine

## Abstract

Branched-chain amino acids (BCAAs)—leucine (Leu), isoleucine (Ile), and valine (Val)—are essential nutrients with significant roles in protein synthesis, metabolic regulation, and energy production. This review paper offers a detailed examination of the physico-chemical properties of BCAAs, their industrial synthesis, and their critical functions in various biological processes. The unique isomerism of BCAAs is presented, focusing on analytical challenges in their separation and quantification as well as their solubility characteristics, which are crucial for formulation and purification applications. The industrial synthesis of BCAAs, particularly using bacterial strains like *Corynebacterium glutamicum*, is explored, alongside methods such as genetic engineering aimed at enhancing production, detailing the enzymatic processes and specific precursors. The dietary uptake, distribution, and catabolism of BCAAs are reviewed as fundamental components of their physiological functions. Ultimately, their multifaceted impact on signaling pathways, immune function, and disease progression is discussed, providing insights into their profound influence on muscle protein synthesis and metabolic health. This comprehensive analysis serves as a resource for understanding both the basic and complex roles of BCAAs in biological systems and their industrial application.

## Introduction

Amino acids (AA), the building blocks of proteins, play pivotal roles in multiple biological processes, from cellular metabolism to muscular physiology. Among these, branched-chain amino acids (BCAAs)—leucine (Leu), isoleucine (Ile), and valine (Val)—hold a distinctive position owing to their unique structural attributes and physiological roles. First identified and isolated in the early twentieth century, BCAAs have since been the focus of extensive research, especially due to their increasing importance in health and disease. As an example, the involvement of BCAAs in cancer (Xu et al. 2023) and diabetes (White et al. 2021) is in focus of many investigations, although their contributions to these diseases remain under debate. In contrast, the metabolic disorder Maple Syrup Urine Disease (MSUD), which is caused by a deficiency in branched-chain keto acid dehydrogenase (BCKDH) complex, is by far better understood (Rodrigues et al. 2022). However, the complexities of these health conditions can’t be easily compared. A key requisite to investigations regarding BCAAs and their metabolites is the use of appropriate analytical methods. In case of BCAAs, differentiation between isomers can be quite challenging in complex samples, especially in case of the diastereomers of Ile and the respective constitutional isomer Leu. As part of this review, state-of-the-art methods and on-going challenges of BCAA analysis are presented.

The similar structure of BCAAs also implies hurdles for their synthesis and purification. As only the L-enantiomers of amino acids are proteogenic, biotechnological processes are favored over chemical synthesis in industrial production of BCAAs due to the stereospecificity of enzymatic reactions. Bacterial strains, such as *C. glutamicum* and *E. coli*, are mainly used for biosynthesis and the processes are constantly sought to be improved, including but not limited to genetic modifications, which are designed to overcome feedback inhibition, increase levels of required substrates and co-factors or enhance transport mechanisms (Xie et al. 2012; Wang et al. 2019b, 2022c; Chen et al. 2022).

As part of the essential amino acids, BCAAs are key components of human diet, both in form of protein-rich food and individual supplements. The uptake and catabolism of BCAAs is very interdependent, as the three AAs share the main transporters and initial metabolizing enzymes, and at the same time they are quite unique due to their catabolism primarily occurring in the muscle rather than the liver. Notably, alterations in the BCAA transport capabilities across plasma membranes through LAT1 were linked to the progression of cancer and diabetes (Kahlhofer and Teis 2022). As Leu is considered one of the main drivers of mTORC1 activation, increased Leu uptake can ultimately lead to increased protein synthesis and cell growth (Goul et al. 2023). Besides being linked to promoting cancer by mTOR hyperactivation, this trait is used in sports nutrition to increase muscle growth or in conditions caused by prior muscles loss (Wolfe 2017; Santos and Nascimento 2019). Altogether, the BCAAs are very multifaceted, and this review serves as a guide through the most crucial aspects of currently investigated applications.

## Chemistry

### Basic description

Each amino acid has a central carbon (α-carbon) linked to a hydrogen atom (H), an amino group (-NH_2_), a carboxyl group (–COOH) and a unique side chain (R).

**Fig. 1:**
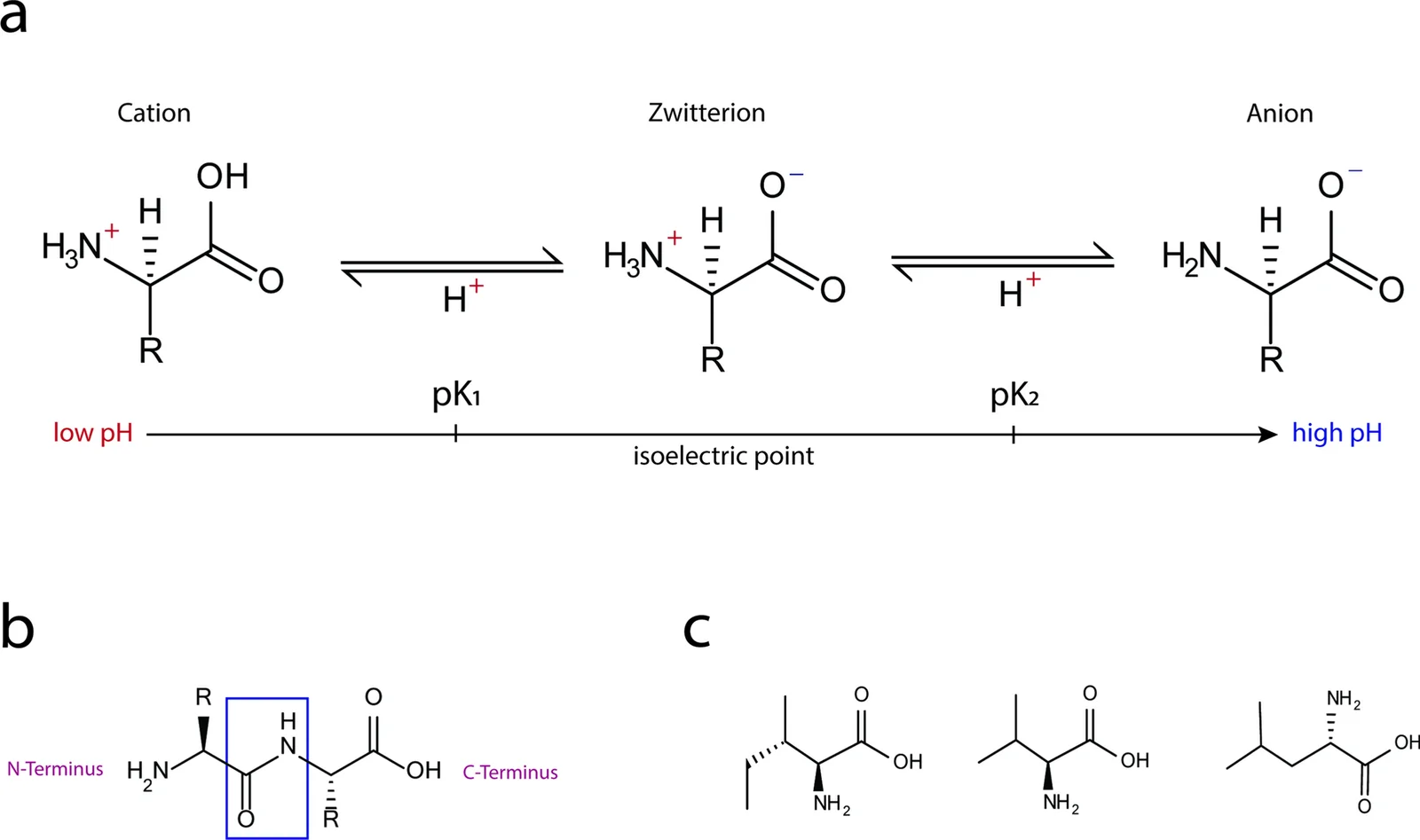
**a** Acid–base reaction leading to differently charged species of AAs depending on pH. At the isoelectric point, the AA exists in its zwitterionic form. **b** A peptide bond is formed between the amino group and carboxyl group of two AAs. The free amino and carboxyl group mark the peptide’s N- and C-terminus, respectively. **c** Molecular structures of Ile, Val and Leu

The side chain defines the AA’s distinct properties, categorizing it as polar, nonpolar, acidic, basic, or aromatic. In water, AAs usually exist in a zwitterionic form, with both positive (− NH_3_^+^) and negative (–COO^−^) charges (Fig. 1a). The carboxyl and amino groups of AAs form peptide bonds (–CONH–), ultimately leading to proteins (Fig. 1b). The characteristics of the AA’s side chains influence protein structure and function.

Ile, Leu and Val are named “branched-chain” because of their aliphatic side chains with a monomethyl-branch (Fig. 1c). Val (C_5_H_11_NO_2_) possesses a five-carbon structure, whereas Ile and Leu (C_6_H_13_NO_2_) have identical molecular formulas, but exhibit isomeric differences due to the position of the branching methyl group at either the 3rd or 4th carbon position.

### Isomerism

#### Constitutional isomers

Constitutional isomers, also known as structural isomers, are compounds that have the same molecular formula but different connectivity or arrangement of atoms. Leu and Ile share a unique relationship as constitutional isomers and differ in the positioning of the branched methyl group. Furthermore, the straight-chain, constitutional isomers of the BCAAs, norleucine and norvaline, do not typically take part in protein synthesis and are hence considered non-proteogenic. However, they can be produced as side-products of BCAA metabolism in *E. coli* and can ultimately be misincorporated into recombinant proteins, causing a major challenge for the pharmaceutical industry. For that reason, they have been and still are under intense investigation (Bogosian et al. 1989; Sycheva et al. 2007; Biermann et al. 2013; Narancic et al. 2019; Martínez-Rodríguez et al. 2020; Mayer et al. 2022). Reitz et al. and Xing et al. reviewed their synthesis pathways and misincorporation in more detail (Reitz et al. 2018; Jin et al. 2019). Garcia et al. showed that glucose and pyruvate (Pyr) pulses combined with oxygen limitation led to the undesired synthesis of norvaline and norleucine in *E. coli* and later demonstrated that modulating the expression of genes involved in the BCAA biosynthetic pathway can reduce the accumulation and consequently misincorporation of these non-canonical BCAAs (García et al. 2021, 2022).

#### Stereoisomers

Stereoisomers are compounds with identical molecular formulas and sequences of bonded atoms, yet their atoms exhibit different spatial arrangements. All alpha AAs, with the exception of glycine, have a chiral center at their alpha carbon. In biological systems, the proteogenic AAs exist predominantly as L-enantiomers, often referred to as S-enantiomers. When considering the BCAAs, Ile presents interesting aspects related to stereoisomerism. Unlike Val and Leu, Ile has an additional chiral carbon in the beta position, leading to four potential stereoisomers. Among these, only the L-isoleucine (2S,3S-configuration) is involved in protein synthesis. L-alloisoleucine (aIle), the 2S,3R-stereoisomer, is typically found in minute quantities in healthy people (Piri-Moghadam et al. 2022b). Further details on this topic will be covered in subsequent sections.

#### Analytical challenges

A common challenge in the chromatographic separation of AAs stems from their inherent polarity. Additionally, most AAs incl. the BCAAs, lack chromophores, which makes direct UV detection especially in complex samples challenging. Therefore, historically, derivatization was the preferred approach to enhance retention in reversed-phase chromatography, particularly when mass spectrometry detection was unavailable. In clinical laboratories, a widespread technique for separating AAs included the use of an ion-pairing agent and a post-column derivatization reagent, like ninhydrin, followed by UV detection for measurement and quantification (Sowell et al. 2011). Alternatively, pre-column derivatization of AAs can be used with subsequent LC-UV analysis allowing for separation of all proteogenic AAs incl. sufficient resolution of BCAAs (Schmidt et al. 2020). Overall, choosing the right method highly depends on the scientific context and the available instrumentation.

The analysis of BCAAs can be intricate due to the presence of both constitutional and stereoisomers. Constitutional isomers, with their differing structural arrangements, and stereoisomers, with their distinct spatial configurations despite identical connectivity, can exhibit overlapping physical and chemical properties. This makes their separation, identification, and quantification challenging using standard analytical methods. Achieving accurate analytical results requires tailored approaches and advanced techniques to discern between these isomeric forms (Biermann et al. 2013; Piri-Moghadam et al. 2022b). The risk of false-positive quantification is not limited to the BCAAs themselves, but also numerous, related metabolites. Therefore, recognizing potential interferences due to isomerism is of high importance (Lai et al. 2022).

As analytical requirements grew more stringent, methodologies evolved, particularly given the significance of BCAAs as biomarkers for serious illnesses. Presently, LC–MS quantification is considered the gold standard because of its efficiency in terms of short run-time, high sensitivity, specificity, and broad dynamic range. Kilgore et al. as well as Piri-Moghadam et al. published similar LC–MS/MS methods without derivatization using the same hydrophilic interaction chromatography (HILIC) column, that allow screening and separation of various AAs incl. constitutional and stereoisomers Leu, Ile and aIle (Piri-Moghadam et al. 2022b, 2022a; Kilgore et al. 2023). Further groups published other quantitation methods by LC–MS without derivatization using an ion pairing reagent on a reversed-phase column (Sowell et al. 2011; Alodaib et al. 2011).

Another recent approach to fast screening of Leu and the sum of Ile/aIle without prior chromatographic separation is flow injection analysis with tandem mass spectrometry followed by multivariate calibration. Compared to univariate calibration using only one MS transition, which leads to high false-positive results, multivariate calibration promises reasonable analytical errors for screening of BCAA levels. Nevertheless, for abnormal concentrations during screening, confirmation with a method including a separation step, as the above-mentioned, is highly recommended (Casas-ferreira et al. 2020).

Furthermore, novel technologies, such as Ion Mobility, were evaluated for analysis of BCAAs by their difference in collision cross section (CCS), representing the effective area, that an ion occupies, while travelling through a gas, and thereby providing insight into its three-dimensional size and shape. A mathematical relationship between experimentally determined CCS and resolving power required for isomer separation was utilized to assess potential resolution of isomers using Ion Mobility. Even though constitutional isomers might be resolved using conventional instruments, the resolution of stereoisomer mixtures remains difficult according to predictions (Dodds et al. 2017).

In proteomics using mass spectrometry (MS), the isomerism of Leu and Ile is an important consideration. In typical bottom-up proteomics using tandem mass spectrometry (MS/MS), Leu and Ile cannot be distinguished based solely on their precursor or fragment ion masses since both have an identical mass. This means that peptides containing these residues often cannot be differentiated based solely on their mass-to-charge ratios. When database searching is used to match MS/MS spectra to peptide sequences, any potential sequence containing either Leu or Ile is considered as a match. This results in multiple potential peptide sequences being considered for a given spectrum, adding ambiguity to peptide identification (Lebedev et al. 2014; Poston et al. 2014; Xiao et al. 2016; Bagal et al. 2017; Zhang et al. 2022).

However, in de novo sequencing of recombinant proteins, unambiguous identification of either Ile or Leu is crucial to accurately characterize therapeutics, in particular given their prevalent presence in proteins and their distinct influence on spatial structures. Historically, peptide sequencing primarily relied on Edman degradation (which could discriminate isobaric Ile and Leu), but this method was progressively replaced by MS-based techniques due to its higher sensitivity, accuracy and ability to handle complex mixtures. Nowadays, multistage fragmentation, preferably by electron capture dissociation (ECD) or electron transfer dissociation (ETD), is applied to generate unique ions for Ile and Leu, but they require instruments to allow MS^3^ fragmentation, such as an Orbitrap. For further details on the fragmentation pattern, several articles may be reviewed (Lebedev et al. 2014; Xiao et al. 2016; Bagal et al. 2017; Zhang et al. 2022).

To date, techniques based on LC–MS are still the most suitable for efficient separation and quantitation of BCAAs and their isomers, whether it is as individual molecules or in form of peptides. State-of-the-art LC–MS/MS methods do not require analyte derivatization even with complex sample matrices and therefore allow for facilitated sample preparation and the use of instrument setups frequently found in modern laboratories. If distinction between these isomers is not relevant, e.g. in case of screenings, flow-injection MS without prior chromatographic separation can be a more time-efficient alternative. An approach suitable for most laboratories, that is capable of separating Ile, Leu and Val, is the use of kits for pre-column derivatization of AAs in combination with LC-UV analysis.

### Solubility

BCAAs are more hydrophobic than many other AAs and hence among the less soluble AAs. Their solubility is pH dependent (Carta and Tola 1996; Pradhan and Vera 1998) and is lowest in water near their pI and highest at pH values significantly above or below their pI (Tseng et al. 2009), due to the presence of electrostatic interactions with water molecules.

This pH-dependent solubility is significant for many applications, such as formulation, separation, and purification processes. BCAAs are consumed as dietary supplements, especially by athletes and their solubility can be vital when formulating products such as powdered drinks, capsules, and tablets. Since solubility affects the bioavailability of AAs, recent studies (Hong et al. 2017b, 2017a, 2018) focused on the development of nanosuspensions or biopolymers to improve the solubilization of BCAAs.

In biotechnological processes, BCAAs may be produced or consumed. Bioavailable derivatives for Ile and Leu, namely their keto acids and N-lactoyl- derivatives, were investigated to replace Ile and Leu in feed formulations used in next generation bioprocesses, aiming at producing monoclonal antibodies (Schmidt et al. 2020, 2021). As an alternative, feeding strategies were specifically developed to circumvent solubility limitations by dissolving Leu in ammonia solution for fermentation with *E. coli* (Beckmann et al. 2017).

In the production of BCAAs, crystallization is frequently used for purification. Understanding solubility is crucial for crystallization design since it dictates the initial amount of solute, that can be dissolved, and the final amount of solute remaining in the solvent after the process. Thus, numerous publications focus on the solubility of BCAAs in various mixtures incl. organic solvents (Ji et al. 2009; Zhang et al. 2014; Do et al. 2021; Wang et al. 2021a, 2023; Xing et al. 2023).

### Industrial Synthesis

#### Upstream

Mammals cannot synthesize BCAAs de novo, rendering them essential. As a result, they must be supplied externally, i.e., through dietary intake or, in case of mammalian cell culture, through media and feeds. To cope with the vast demand for BCAAs, industrial synthesis methods have been developed, primarily harnessing the production capabilities of bacterial strains (D’Este et al. 2018). The Gram-positive bacterium, *Corynebacterium glutamicum*, stands out in this respect as one of the main workhorses for industrial BCAA production. This bacterium not only plays a pivotal role in BCAA production but is also employed in the synthesis of other AAs like lysine, glutamate, threonine, and serine (Zhang et al. 2020b). Other bacterial strains, such as *E. coli* and *Pantoea ananatis*, are also used for AA production (Hook et al. 2022; Wang et al. 2022b; Pan et al. 2022).

*C. glutamicum* naturally synthesizes BCAAs efficiently due to its inherent metabolic pathways and enzymes. Through genetic engineering and strain enhancement, its BCAA production capacity has been amplified and this strain is now renowned for its durability, managing to thrive in challenging industrial settings, including high substrate concentrations and low pH. This combination of natural metabolic prowess, resilience, safety, and genetic adaptability makes it ideal for large-scale BCAA production. The use of *C. glutamicum*, but also *E. coli,* to produce BCAAs and other AAs has been reviewed in a vast number of publications (Park and Lee 2010; Dong et al. 2011; Oldiges et al. 2014; Amorim Franco and Blanchard 2017; Wang 2019; Wang et al. 2019a; Liang et al. 2021; Gao et al. 2021; Yu et al. 2021; Kranz et al. 2022). In their review, Park and Lee (2010) elaborated on differences in BCAA biosynthesis between *C. glutamicum* and *E. coli*, but the overall biosynthetic pathways, their regulation and strategies to improve production of individual BCAAs are very similar. As this review aims to provide a general overview of the process and improvement strategies and because it can be mainly applied to other strains such as *E. coli*, the following explanations are based on literature involving *C. glutamicum*.

**Fig. 2:**
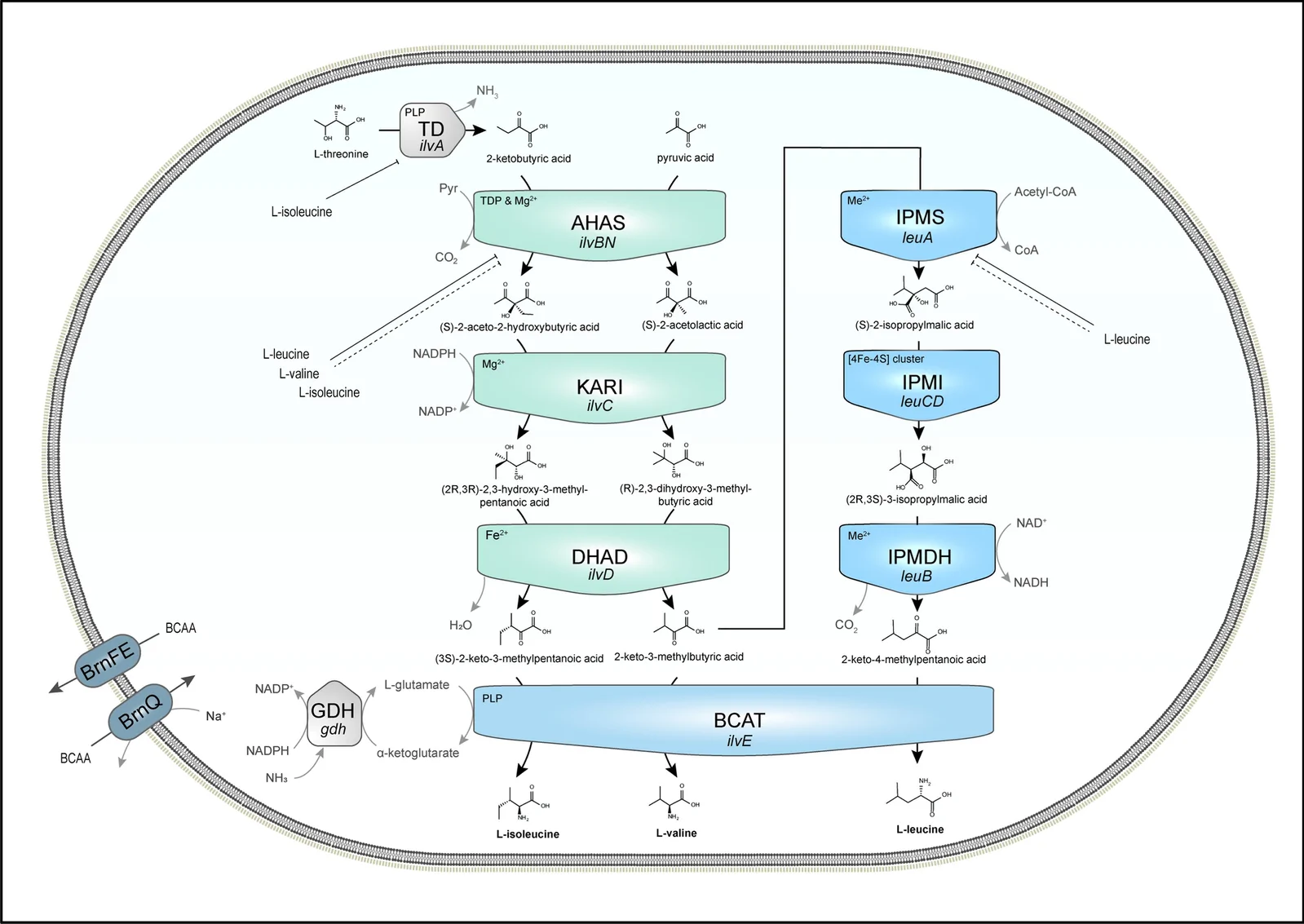
Pathway of Ile, Leu and Val biosynthesis in *C. glutamicum* starting from precursors 2-ketobutyrate (formed from L-threonine by Threonine dehydratase/deaminase (TD)) and pyruvate. Acetohydroxy acid synthase (AHAS) then catalyzes the condensation of pyruvate to form acetolactate, a precursor for L-valine, and acetohydroxybutyrate for L-isoleucine. Ketol-acid reductoisomerase (KARI) reduces acetolactate and acetohydroxybutyrate into their corresponding 2,3-dihydroxy derivatives. Dihydroxyacid dehydratase (DHAD) catalyzes the subsequent dehydration of these dihydroxy acids to yield the alpha keto acids of Val and Ile. 2-Keto-3-methylbutyric acid is elongated and converted into 2-keto-4-methylpentanoic acid through the action of 2-isopropylmalate synthase (IPMS), which adds an acetyl group, followed by isomerization by 2-isopropylmalate isomerase (IPMI) and oxidative decarboxylation by 3-isopropylmalate dehydrogenase (IPMDH), resulting in the formation of the keto acid precursor for L-leucine. Ultimately, the BCAAs are obtained by transamination of the corresponding keto acids by Branched-Chain Aminotransferase (BCAT), using glutamate as amino group donor. Glutamate dehydrogenase (GDH) regenerates glutamate under consumption of ammonia and NADPH. BrnQ and BrnFE are transporters for BCAA influx and efflux, respectively. Terminating symbol of lines indicates feedback inhibition (solid lines) or transcription attenuation (dotted lines)

Liang et al. (2021) extensively reviewed the enzymes involved in the biosynthetic pathway of the BCAAs focusing on their chemical mechanisms, also highlighting the differences between organisms. The native process in *C. glutamicum* is simplified in Fig. 2 (modified from Liang et al. (2021) and Yu et al. (2021)). Generally, Val and Ile undergo similar enzymatic processes, but they originate from distinct precursors: 2-ketobutyric acid (KB) for Ile and Pyr for Val. While Pyr is mainly provided through glycolysis, KB is derived from L-threonine under formation of ammonia by catalysis of the pyridoxal phosphate (PLP)-dependent enzyme threonine dehydratase (TD, EC 4.3.1.19, gene: *ilvA*). In multiple steps, threonine is originally derived from oxaloacetate, which is formed from the glycolytic intermediate phosphoenolpyruvate in *C. glutamicum* (Yu et al. 2021). Hence, all BCAAs can be produced from glucose as starting material to the biosynthetic process.

As the first common step in BCAA biosynthesis, acetohydroxy acid synthase (AHAS, EC 2.2.1.6, gene: *ilvBN*) catalyzes the condensation of Pyr with either KB or another molecule of Pyr to form the two intermediates (2S)-2-acetyl-2-hydroxybutyrate (Ile precursor) or (2S)-2-acetolactate (Val precursor), respectively. Notably, while other bacterial strains, such as *E. coli*, possess three isoenzymes of AHAS, *C. glutamicum* contains only one. The reaction releases one molecule of CO_2_ and requires thiamine diphosphate (TDP) and Mg^2+^ as cofactors. TD and AHAS were found to be susceptible to feedback inhibition by accumulation of Ile or all three BCAAs, respectively (Eggeling et al. 1987; Leyval et al. 2003; Elišáková et al. 2005; Guo et al. 2015). Furthermore, AHAS was found to be controlled by transcriptional attenuation through the BCAAs (Morbach et al. 2000). For that reason, identifying feedback-resistant strains was a crucial part in refinement of BCAA biosynthesis. Ketol-acid reductoisomerase (KARI, EC 1.1.1.86, gene: *ilvC*) converts the two respective intermediates into 2,3-dihydroxycarboxylic acids by migration of an alkyl group followed by a reduction by nicotinamide adenine dinucleotide phosphate in its reduced form (NADPH). KARI also requires Mg^2+^ to function. Under release of a water molecule, dihydroxyacid dehydratase (DHAD, EC 4.2.1.9, gene: *ilvD*) converts the 2,3-dihydroxycarboxylic acids into the respective branched-chain keto acids (BCKA). DHAD, an iron-sulfur cluster enzyme, needs divalent ions, such as ferrous ion, for catalysis (Liang et al 2021).

Leu is synthesized through further sequential steps from 2-keto-3-methylbutyric acid, the final intermediate in Val production. In the initial step, this molecule reacts with acetyl-CoA (Ac-CoA) to form (S)-2-isopropylmalate releasing CoA in a condensation reaction, which is catalyzed by 2-Isopropylmalate synthase (IPMS, EC 2.3.3.13, gene: *leuA*), an enzyme able to utilize various metal ions as cofactor. IPMS is regulated by feedback inhibition and transcriptional attenuation through Leu accumulation, but variants with increased feedback resistance were implemented combined with replacement of the native *leuA* promoter to circumvent attenuation (Vogt et al. 2014; Huang et al. 2017). Subsequent isomerization of (S)-2-isopropylmalate results in the formation of (2R,3S)-3-isopropylmalate. This reaction involves the [4Fe–4S] cluster protein isopropylmalate isomerase (IPMI, EC 4.2.1.33, gene: *leuCD*). By oxidation of the α-hydroxy group and subsequent decarboxylation, 3-isopropylmalate dehydrogenase (IPMDH, EC 1.1.1.85, gene: *leuB*) converts the previous intermediate into 4-methyl-2-ketopentanoate, while reducing nicotinamide adenine dinucleotide (NAD^+^) to NADH. Ultimately, the production processes of all three BCAAs converge on a final enzymatic stage, the reversible transamination by branched-chain aminotransferase (BCAT, EC 2.6.1.42, gene: *ilvE*) in presence of PLP. In this reaction, the amino group of L-glutamate is transferred to the branched-chain keto acids, resulting in the respective BCAA and α-ketoglutarate (Liang et al 2021). Glutamate is regenerated by glutamate dehydrogenase (GDH, EC 1.4.1.4, gene: *gdh*) from α-ketoglutarate and ammonia under consumption of NADPH (Hasegawa et al. 2012). Overall, the intersection in the biosynthesis pathways of all three BCAAs, namely the overlapping enzymatic steps as well as the use of Val’s α-keto acid as precursor for Leu production, highlights the challenge in selectively producing the individual BCAAs.

Yu et al. (2021), Wang et al. (2018) and Liu et al. (2021) reviewed recent advances in BCAA production by *C. glutamicum* in vast detail, but in the following the most important approaches to engineering this strain will be explained. Notably, very similar strategies are applied to enhance BCAA biosynthesis in other strains, such as *E. coli*. Besides modulating expression of the endogenous enzymes directly involved in biosynthesis of BCAAs to direct the fluxes towards hyperproduction of individual BCAA, *C. glutamicum* was genetically modified on a variety of targets. As an example, expression of enzymes unique to the synthesis of Ile and Leu, such as TD or IPMS, can be reduced to channel metabolism towards Val production. Overexpression of the enzymes involved in BCAA biosynthesis increases their overall activity and BCAA production. However, native bacterial strains, such as *C. glutamicum*, offered potential for improvement including, but not limited to transport processes, substrate availability and resistance to feedback inhibition (Chen et al. 2015). Xie et al. (2012) showed that the deletion of uptake carrier BrnQ and overexpression of export carrier BrnFE increased the net efflux of Ile and thereby increased the production titer by approx. 50% in fed-batch. A side benefit of increasing efflux is the decreased feedback control of Ile towards its biosynthesis due to decreased intracellular concentrations. Yin et al. (2013) investigated the combined overexpression of BrnFE and global regulator Lrp and obtained a 72% increase in Ile production compared to the control strain.

Feedback resistance of specific enzymes in the BCAA biosynthetic pathways was further enhanced upon identification of feedback resistant enzyme variants. Huang et al. (2017) and Vogt et al. (2014) identified similar IPMS mutants (R529H, G532D and L535V; R529H and G532D) for Leu production in *C. glutamicum*. Both groups combined the integration of these mutants with deletion of the repressing regulator Ltbr, which led to increased expression of *leuBCD*, in order to enhance Leu production. Furthermore, in these studies, concentrations of precursors, such as Ac-CoA or Pyr, were increased by inactivating citrate synthase, alanine aminotransferase or lactate dehydrogenase. Vogt et al. (2015) also succesfully implemented a mutant of TD with increased feedback resistance for the improved production of Ile. AHAS is another enzyme susceptible to feedback control by all BCAAs. Elišáková et al. (2005) performed site-directed mutagenesis on this enzyme increasing its feedback resistance and thereby enhanced Val production in *C. glutamicum*.

Pyr availability, which is an initial substrate to the biosynthesis of all three BCAAs, was the center of many investigations, e.g., those carried out by Buchholz et al. (2013) and Blombach et al. (2007) with the ultimate goal of reducing competitive Pyr consumption, e.g., by the tricarboxylic acid (TCA) cycle. While Blombach et al. deleted the gene *aceE*, that encodes the E1 subunit of Pyr dehydrogenase (PDH) complex, Buchholz et al. gradually reduced and thereby optimized the activity of PDH complex by substitution of the gene’s promoter with those of the *dapA* library, which allowed for gradually differing promoter activity. On this basis and in combination with overexpression of *ilvBNCE*, both groups achieved increases in Pyr and consequent Val production. Radmacher et al. (2002) chose an alternative approach to block Pyr decarboxylation through the PDH complex. In their study, the *panBC* gene was deleted to decrease pantothenate availability for CoA production, which is essential to the decarboxylation process of Pyr via PDH. Also, the last Val intermediate 2-keto-3-methylbutyric acid is a substrate to pantothenate biosynthesis and thereby the increased availability of the keto acid as a result of *panBC* deletion also might have contributed to the increased Val production (Radmacher et al. 2002). Besides PDH, activities of other Pyr-converting enzymes, such as alanine aminotransferase (gene: *alaT*), alanine-valine transaminase (gene: *avtA*), lactate dehydrogenase (gene: *ldhA*), pyruvate carboxylase (gene: *pyc*), pyruvate:quinone oxidoreductase (gene: *pqo*) were reduced to successfully increase Pyr levels and Val production (Blombach et al. 2008; Huang et al. 2017) (Wang et al. 2020). As Leu biosynthesis requires Ac-CoA, reducing its availability by interventions, such as PDH deletion, are counterproductive, when Leu production is desired. On the contrary, Ac-CoA supply was considered limiting and rather needed to be enhanced (Vogt et al. 2014). Increasing Pyr availability for ultimate Leu production was then rather achieved by deletion of genes expressing other Pyr-consuming enzymes, such as *pyc* and *alaT* (Wang et al. 2020). In order to increase Ac-CoA availability, citrate synthase activity (gene: *gltA*) was reduced by integration of a terminator in front of its gene, while Ac-CoA synthase (gene: *acs*) and deacetylase (gene: *cobB*) were overexpressed paired with acetate supplementation, in order to enhance Ac-CoA synthesis and to reduce its consumption via competing pathways, such as the TCA cycle (Wang et al. 2022c).

The biosynthetic process of BCAAs also requires sufficient supply of reducing equivalents. In native *C. glutamicum*, two NADPH are consumed during the synthesis of Ile, Leu and Val, starting from KB or Pyr, respectively, each one for the reduction catalyzed by KARI and the glutamate regeneration by GDH, as shown in Fig. 2. As glycolysis, which provides the initial substrate Pyr for overproduction of BCAAs, generates two NADH per mol of glucose, among others, Hasegawa et al. (2012) hypothesized a redox imbalance limiting the biosynthetic process. The optimization of the redox balance was approached with different strategies. For the ultimately increased production of Val, Bartek et al. (2010) deleted the gen *pgi*, encoding phosphoglucoisomerase, to increase the flux and thereby the generation of NADPH in the pentose phosphate pathway. Shi et al. (2013) increased the intracellular NADPH levels and consequently the Ile production by overexpression of the genes *zwf* and *ppnK*, encoding glucose-6-phosphate dehydrogenase and NAD^+^ kinase, respectively. While these approaches increased the production of NADPH, Hasegawa et al. (2012, 2013) and Wang et al. (2019b) changed the enzyme specificity for NADPH to NADH by modification of KARI and introduction of NAD^+^-dependent Leu dehydrogenase (LeuDH, gene: *leuDH*) from *Lysinibacillus sphaericus* instead of endogenous BCAT for the amination process. LeuDH has varying affinities for the three BCAAs, which offers another advantage in Leu biosynthesis. While avoidance of Ile by-product formation is straight forward by deletion of *ilvA* expressing TD, preventing Val accumulation is more challenging, as the only step in the biosynthesis, that separates the initital Leu substrate 2-keto-3-methylbutyric acid from conversion to Val, is the transamination by BCAT, which is also required in the last step of Leu production. However, LeuDH specificity for Leu turned out to be superior than for Val and therefore BCAT replacement by LeuDH resulted in decreased Val by-product formation improving the composition of the final product (Wang et al. 2019b).

Overall, many enzymes as well as the precursors of BCAAs have been focal points in recent refinement efforts, highlighting the extensive research and understanding of AA producing organisms, such as *C. glutamicum* and *E. coli*. More recently, omics studies have been used to identify potential limitations or possibilities for improvement. As an example, Ma et al. (2018) performed a genomic study on *C. glutamicum* comparing a reference strain with strains, which were developed for Leu or Val hyperproduction by random mutagenesis. They identified genes, such as *leuA*, global regulator *lrp* and *brnFE*, which had mutations or additional copies benefiting BCAA hyperproduction. Similarly, Zhang et al. (2018a) performed a comparison with a Val overproducing strain on a transcriptomic and proteomic level. Their study highlighted the upregulation of key genes in the Val biosynthesis pathway. Additionally, upregulation of genes in pentose phosphate and TCA pathway indicated the importance of NADPH and ATP availability for hyperproduction of BCAAs. Both groups identified beneficial alterations in genes, that ultimately led to Pyr accumulation, the initial substrate for Val biosynthesis. Ultimately, the identified targets in these omics studies were in good agreement with achievements from previous genetic engineering approaches.

#### Downstream

As an alternative to fermentation processes, AAs can be obtained from the hydrolysis of protein-containing materials, such as soybeans, but this process remains a minor fraction of total AA synthesis. These processes are carried out in the presence of a strong acid or base and the AAs are then isolated through precipitation. In this case, understanding how pH influences AA solubility in water is vital for intelligently designing subsequent downstream operations (Tseng et al. 2009).

Independently of the source of crude product, whether it is through fermentation or hydrolysis of protein-containing material, producing BCAAs is a challenging process, particularly when the objective is to obtain a purified product, devoid of contamination from the other two BCAAs. The downstream processing that follows fermentation is designed to meticulously separate, purify, and concentrate each BCAA, ensuring minimal cross-contamination. Over the past decades, several approaches have been developed: different pH values, temperatures, and mixtures of organic solvents.

Following the fermentation phase and initial separation of microbial cells, the real challenge begins. The broth, enriched with BCAAs and other metabolic products, must undergo multiple purification steps. Given the structural similarity of the BCAAs, their separation from one another is non-trivial, as indicated in various patents (Kurosawa et al. 2005, patents: US3960942A, US4263450A, JPH10237030A, CN105274179B, CN108707083B, CN108285912B, CN103819352A, CN110372528B).

Selective crystallization has proven to be an effective strategy at industrial scale, because the three BCAAs exhibit unique solubilities and crystallization behaviors under different pH environments. For example, at pH values of 1 to 2, Leu crystallizes first, followed by crystallization of Ile under more stringent conditions, i.e. cooling from approx. 70 °C in presence of concentrated hydrochloric acid. Ultimately, through processes like esterification and fractional distillation, Val can be obtained from the remaining mother liquor (patent: KR830001464B1). Such pH-driven fractional crystallization takes advantage of the differential solubilities of BCAAs, allowing for their sequential separation. Alternatively, other methods have been investigated, such as high-pressure crystallization. Pressure crystallization is advantageous in that pressure is transmitted to the sample solution instantly and thermal decomposition of the solute on crystallization by heating is avoided. In such crystallization processes, solubility at high pressure is an important basic property, necessitating accurate solubility information (Matsuo et al. 2002).

Moreover, the use of specific metal-ion complexes offers a distinct route for separating BCAAs. Methods that rely on the differential solubilities of Leu and Ile when forming complexes with copper in methanol have been refined. Improved methods involve fractional crystallization using copper or nickel salts of these AAs. This process alternates between relatively low and high pH-values in the acidic range, ensuring effective separation (patent: US3960942A).

Ion-exchange chromatography is another pillar in the purification regimen. By capitalizing on the slightly different charges of BCAAs at specific pH values, chromatographic techniques can help to separate them based on their affinities for the charged resin in the column. Since this method can achieve a significant degree of purification, it is frequently used as a polishing step, ensuring the product is free from other contaminants and other BCAAs.

Subsequent processes, such as decolorization with activated carbon, fine filtration, and drying, ensure that the final product is of the highest purity. The configuration of a desired crystal can be modified using particular crystallization techniques. Both thermodynamic and kinetic factors play a role in crystallization, and the introduction of different conditions, such as solvents or additives, can lead to the formation of diverse polymorphic structures (Liu and Li 2008). Of course, rigorous quality control measures and analytics are paramount throughout these stages. They are needed to confirm the absence of any BCAA cross-contamination and ascertain that the final product aligns with its intended quality and purity benchmarks.

In summary, while the structural similarities of BCAAs pose a challenge in their purification, a combination of crystallization techniques, ion-exchange chromatography, and meticulous pH manipulations ensure the efficient separation and purification of each BCAA, achieving products of the highest purity.

## Uptake, metabolism and fate of BCAAs

### Uptake/compartmentalization

In the human genome, more than 60 transporters are known to be involved in the transport of AAs (Kandasamy et al. 2018). They differ, for example, in substrate specificity, transport mechanism as well as tissue and subcellular distribution (Table 1). This chapter initially describes AA intake, focusing on processes involving BCAAs during intestinal absorption and transport to the liver. Subsequently, transporters and mechanisms applying to other tissues are discussed.

**Table 1:** BCAA-related transporters including the solute carrier families, protein names, AA substrates and transport mechanism (modified from Fotiadis et al. 2013; Kandasamy et al. 2018))

Human gene name	Protein Name	Predominant AA substrates	Mechanism
SLC6A15	B^0^AT2	BCAA	Co-transport of BCAA with Na^+^
SLC6A17	B^0^AT3	AA^0^	Co-transport of BCAA with Na^+^ and Cl^−^
SLC6A19	B^0^AT1	AA^0^	Co-transport of AA^0^ with Na^+^
SLC7A5	LAT1	Large AA^0^	Exchange with AA^0^, such as Gln
SLC7A6	y^+^LAT2	AA^+^, large AA^0^	Co-transport of Na^+^ with large AA^0^ in exchange with AA^+^
SLC7A7	y^+^LAT1	AA^+^, large AA^0^	Co-transport of Na^+^ with large AA^0^ in exchange with AA^+^
SLC7A8	LAT2	AA^0^	Exchange with AA^0^, such as Gln
SLC7A9	b^0,+^AT	AA^+^, large AA^0^	Exchange of AA^+^ with AA^0^
SLC43A1	LAT3	BCAA	Facilitated diffusion
SLC43A2	LAT4	BCAA	Facilitated diffusion
SLC25A44	Mitochondrial BCAA Carrier	BCAA	n. a

Their protein name describes the transport system indicative of their substrate specificity and transport mechanism. “LAT” indicates the transport system L for large AA^0^, while “y^+^” refers to the additional transport of AA^+^. “b” represents Na^+^-independent transporters of broad specificity accepting AA^0^, while the additional superscript “^+^” indicates the transport of AA^+^. The upper case “B” indicates Na^+^-dependency

BCAAs can be ingested either in their “pure” form as a supplement or in the form of proteins, which are eventually hydrolyzed to AAs. In case of protein ingestion, proteins are denatured in the acidic stomach environment and digested by pepsin into polypeptides. The partially digested proteins are subjected to further digestion in the small intestine by proteolytic enzymes released from the pancreas, such as trypsin, chymotrypsin, carboxypeptidase and elastase, leading to AAs, di- and tripeptides. In the duodenum, brush border enzymes, aminopeptidases and dipeptidases, break down the remaining peptides into individual AAs (Loveday 2022).

Free AAs are then taken up by intestinal cells. This process is part of the broader mechanism of secondary active transport, which leverages the energy stored in the gradient of another molecule, e.g. Na^+^, to facilitate the transport of AAs into intestinal cells. Various transporters play a role in these processes, aiming to transfer AAs into epithelial cells, before they are eventually released into the bloodstream on the basolateral side. Different AA transporters in the intestinal membrane have specificity for different AA groups (e.g., neutral (AA^0^), anionic (AA^−^), cationic (AA^+^)). The Na^+^-dependent B^0^AT1 (encoded by the SLC6A19 gene) facilitates the uptake of AA^0^, including BCAAs. AA^0^ have a further important role in assisting transport of AA^+^ by means of antiport through solute carrier (SLC) b^0,+^AT (SLC7A9), i.e. transport AA^+^ into the intestinal cells in exchange for AA^0^. This phenomenon of concurrent presence of symporters and antiporters with overlapping substrate specificity results in competition of AA uptake. Some AAs might compete for the same transporters. If the diet is particularly rich in one AA, it might reduce the uptake of another AA, that shares the same transporter. This phenomenon underscores the importance of a balanced intake of AA. Di- and tripeptides, which of course can also contain BCAAs, are typically transported by PEPT1 (encoded by the SLC15A1 gene) in symport with protons, allowing these peptides to be broken down by cytoplasmic peptidases (Fotiadis et al. 2013; Kiela and Ghishan 2016; Bröer and Gauthier-Coles 2022).

Absorbed AAs are then transported into the bloodstream on the cell’s basolateral side. Again, different transport mechanisms are involved for different groups of AAs. LAT2 (encoded by the SLC7A8 gene) as well as the uniporter LAT4 (SLC43A2 gene) are believed to handle the transport of AA^0^, including BCAAs (Fotiadis et al. 2013; Zhang et al. 2018b; Bröer and Gauthier-Coles 2022). The efflux via LAT2 operates in antiport with another AA^0^. Also, AA^0^, here along with Na^+^, serve as substrate for antiport to enable efflux of AA^+^, e.g. arginine, by y^+^LAT1 (gene SLC7A7) (Rotoli et al. 2020). Once more, this underscores competitiveness of the AA uptake. Once in the blood stream, the AAs are transported into the liver through the portal vein (Kohlmeier 2015; Kiela and Ghishan 2016).

BCAAs are transported into the liver through LAT3 (SLC43A1) (Babu et al. 2003; Fukuhara et al. 2007). Unlike some other AAs, BCAAs are not extensively metabolized in the liver. BCAT activity in the liver is low and thus BCAAs bypass significant regulation in this organ before entering the bloodstream, where they become available for uptake by different tissues. In contrast, the corresponding BCKAs, which are extensively produced in muscles, are taken up in the liver and irreversibly oxidized through branched-chain keto acid dehydrogenase (BCKDH) complex (Suryawan et al. 1998; Paulusma et al. 2022).

Besides uptake processes, other transporters and mechanisms related to BCAAs play an important role in human physiology. While LAT1 (gene: SLC7A5) does not play a major role in dietary uptake of AA in the intestine, it is, along with LAT2, mainly responsible for the transport of BCAAs across the plasma membrane. Both transporters are found in various tissues and are covalently bound to 4f2hc (aka. CD98, gene: SLC3A2) by a disulfide bridge. LAT1, which is the most prominent transporter of BCAAs, is a Na^+^- and pH-independent transporter, that exchanges BCAAs (and aromatic AA^0^) in 1:1 antiport with AAs such as histidine, tyrosine and glutamine (Gln). For instance, an increased biosynthesis or influx of intracellular Gln through SNAT2 results in an increased exchange of AAs by LAT1. This antiport mechanism plays an important role in activation of the mammalian target of rapamycin (mTOR) complex 1 (Dodd and Tee 2012). LAT1 is related to various types of cancer (hyperactivation of mTOR through AA excess) and other diseases. Therefore, it is the most investigated among the transporters of BCAAs (Napolitano et al. 2015, 2017; Bröer and Bröer 2017; Singh and Ecker 2018; Scalise et al. 2018; Häfliger and Charles 2019; Puris et al. 2020; Hewton et al. 2021; Nachef et al. 2021; Saito and Soga 2021; Kahlhofer and Teis 2022).

Less extensively researched BCAA transporters are LAT3 (gene: SLC43A1) and LAT4 (gene: SLC43A2), which are AA facilitated diffusers and not exchangers. LAT3 was mainly described in liver, skeletal muscle and pancreas (Babu et al. 2003), while LAT4 is found in various other tissues and contributes to basolateral release of AA^0^ in intestinal cells (Bodoy et al. 2013). Furthermore, B^0^AT2 and B^0^AT3 are known, but also comparably less researched transporters of neutral AA^0^ including BCAAs (Takanaga et al. 2005; Fairweather et al. 2015).

In terms of subcellular transport, trafficking across the mitochondrial membrane was found to be mediated by SLC25A44 (Yoneshiro et al. 2019, 2021; Walejko et al. 2021). The transport of BCKA across the mitochondrial or plasma membrane is possible through monocarboxylate transporters MCT1 (gene SLC16A1) and MCT4 (gene SLC16A4) (Hewton et al. 2021). These mechanisms allow for further catabolism of BCAA and BCKA in the mitochondria.

### Catabolism

**Fig. 3:**
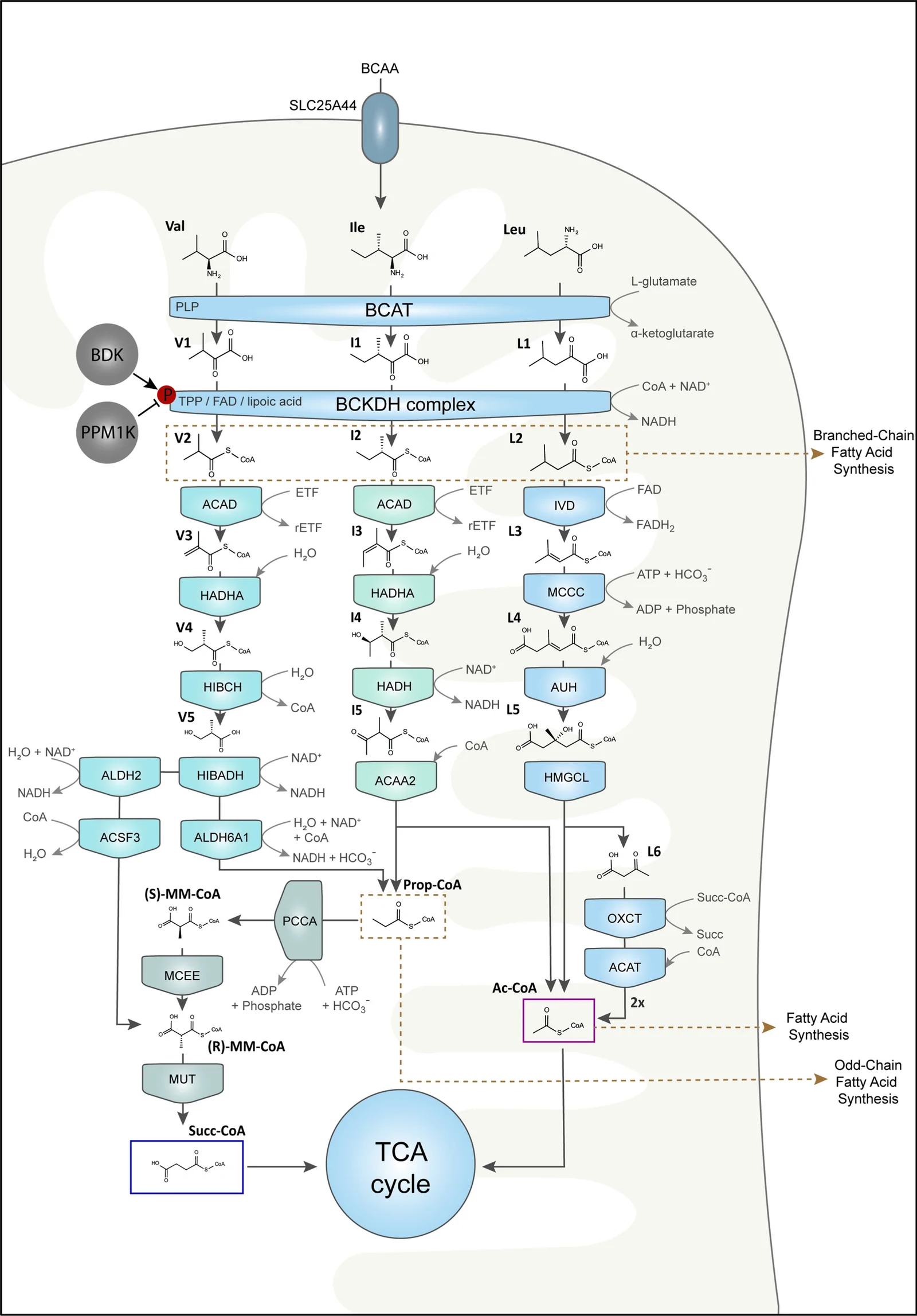
Catabolism of BCAAs in the mitochondria starting with transamination by BCAT and oxidative decarboxylation by BCKDH complex. Ultimately, BCAAs are broken down into acetyl-CoA or succinyl-CoA (intermediate: propionyl-CoA), which can be integrated into the TCA cycle. Besides the CoA derivatives resulting from decarboxylation by BCKDH complex, acetyl-CoA and succinyl-CoA can be used for fatty acid synthesis. Enzymes: BCAT, Branched-chain amino acid transaminase; BCKDH, Branched-chain keto acid dehydrogenase; IVD, isovaleryl-CoA dehydrogenase; MCCC, β-methylcrotonyl-CoA carboxylase; AUH, methylglutaconyl-CoA hydratase; HMGCL, 3-hydroxy-3-methylglutaryl-CoA lyase; OXCT, 3-oxoacid CoA-transferase; ACAT, acetyl-CoA C-acetyltransferase; ACAD, 2-methylacyl-CoA dehydrogenase; HADHA, enoyl-CoA hydratase; HADH, 3-hydroxy-2-methylbutyryl-CoA dehydrogenase; ACAA2, acetyl-CoA acyltransferase; PCCA, propionyl-CoA carboxylase; MCEE, methylmalonyl-CoA epimerase; MUT, methylmalonyl-CoA mutase; HIBCH, 3-hydroxyisobutyryl-CoA hydrolase; HIBADH, 3-hydroxyisobutyrate dehydrogenase; ALDH2, aldehyde dehydrogenase; ALDH6A1, methylmalonate-semialdehyde dehydrogenase; ACSF3, methylmalonyl-CoA synthetase; BDK: BCKDH kinase; PPM1K, protein phosphatase 1 K (Mg^2+^/Mn^2+^ dependent); (r)ETF, (reduced) electron-transfer flavoprotein

The metabolism of BCAAs (Fig. 3) represents a complex sequence of enzymatic steps, starting with initial, reversible transamination, followed by the rate-limiting, irreversible decarboxylation and ending with integration into the tricarboxylic acid cycle, where the compounds contribute to the generation of NADH and eventually ATP.

#### Transamination of BCAAs

The foundational step in the metabolism of BCAAs is transamination, where the amino group is transferred from the BCAA to an acceptor keto acid, leading to the formation of branched-chain α-keto acids (BCKA) (and the corresponding AA). This reaction is executed by the PLP-dependent enzyme BCAT (EC: 2.6.1.42). When BCAAs undergo transamination mediated by BCAT, Leu gives rise to α-ketoisocaproate (**L1**), Ile produces α-keto-β-methylvalerate (**I1**), and Val forms α-ketoisovalerate (**V1**). Most literature sources state, that the removed amino group is transferred to α-ketoglutarate, resulting in the synthesis of glutamate (Harris et al. 2005; Walejko et al. 2021), but there is also evidence that the exchange e.g. in CHO cells mainly occurs between BCAAs and the respective other BCKAs (Schmidt et al. 2020).

BCAT exists in both mitochondrial (BCATm or BCAT2) and cytosolic (BCATc or BCAT1) forms. Even though substrate specificity for BCAAs is similar, the role of the two variants is different in their respective subcellular compartments and expressing tissues. While BCATm is ubiquitously expressed except in the liver, BCATc is only found in brain, ovary and kidney (Hall et al. 1993; Nong et al. 2022). The expression of BCATc in its limited tissues is attributed to distinct roles, e.g. in the brain, it is thought to be responsible for replenishing glutamate, an excitatory neurotransmitter, which itself serves as a precursor for inhibitory neurotransmitter γ-aminobutyric acid (GABA) (Cole et al. 2012). Generally, in the mitochondria, the BCKA formed through BCATm are immediate substrates for subsequent oxidative decarboxylation and further metabolism.

#### Oxidative decarboxylation and the role of BCKDH complex

In the mitochondria, the BCKDH complex facilitates the oxidative decarboxylation of the aforementioned α-keto acids. With similar function and structure as PDH and α-ketoglutarate dehydrogenase (OGDH), this complex is an assembly of multiple enzymes: E1 (EC 1.2.4.1, complex specific dehydrogenase), E2 (EC 2.3.1.12, transacylase), and E3 (EC 1.8.1.4, dihydrolipoamide dehydrogenase). Notably, the activity of the BCKDH complex is regulated through phosphorylation on Ser293 on the E1 subunit by the branched-chain keto acid dehydrogenase kinase (BDK or BCKDK) and dephosphorylation by the phosphatase PPM1K, also referred to as PP2Cm (Lu et al. 2009). The phosphorylated form of the BCKDH complex is inactive, while the dephosphorylated form is active. This regulatory control is essential for maintaining AA homeostasis in the body.

For the BCKDH complex to function, it relies on various cofactors, including thiamine pyrophosphate (TPP) for E1, coenzyme A (CoA) and lipoic acid for E2 as well as flavin adenine dinucleotide (FAD) and nicotinamide adenine dinucleotide (NAD^+^) for E3. The resulting products from this decarboxylation process are CoA esters: the keto acids of Leu (**L1**), Ile (**I1**) and Val (**V1**) are converted to isovaleryl-CoA (**L2**), (S)-2-methylbutanoyl-CoA (**I2**), and isobutyryl-CoA (**V2**), respectively (Paxton et al. 1986; Yeaman 1989; Harris et al. 1997).

#### Subsequent metabolic steps post-decarboxylation

Upon decarboxylation, several sequential steps lead to formation of **Ac-CoA** and/or succinyl-CoA (**Succ-CoA**), which can be utilized in the TCA cycle. Multiple intermediates are connected to other metabolic pathways, especially **Ac-CoA** has multiple functions (Shi and Tu 2015). However, in the following section, only the pathways connecting BCAA metabolism and the TCA cycle will be described.

Leu Metabolism:

**L2** undergoes a series of reactions, first being dehydrogenated to β-methylcrotonyl-CoA (**L3**) by isovaleryl-CoA dehydrogenase (IVD, EC: 1.3.8.4). The carboxylation of **L3** subsequently yields β-methylglutaconyl-CoA (**L4**) through the biotin-dependent β-methylcrotonyl-CoA carboxylase (MCCC, EC: 6.4.1.4). Following this, methylglutaconyl-CoA hydratase (AUH, EC: 4.2.1.18) mediates the hydration to produce (S)-3-hydroxy-3-methylglutaryl-CoA (**L5**, HMG-CoA). Finally, **L5** is cleaved by HMG-CoA lyase (HMGCL, EC: 4.1.3.4) into acetoacetate (**L6**) and acetyl-CoA (**Ac-CoA**), which can directly be utilized in TCA cycle. The formed **L6** can be used to generate more **Ac-CoA** by means of 3-oxoacid CoA-transferase (OXCT, EC: 2.8.3.5) and Ac-CoA C-acetyltransferase (ACAT, EC: 2.3.1.9).

Ile Metabolism:

**I2** is converted to trans-2-Methylbut-2-enoyl-CoA (**I3**) by 2-methylacyl-CoA dehydrogenase (ACAD, EC: 1.3.8.5). Consequently, hydration by enoyl-CoA hydratase (HADHA, EC: 4.1.2.17) results in (S)-3-Hydroxy-2-methylbutyryl-CoA (**I4**), which is followed by dehydrogenation by 3-hydroxy-2-methylbutyryl-CoA dehydrogenase (HADH, EC: 1.1.1.35/1.1.1.178) to form 2-Methyl-3-acetoacetyl-CoA (**I5**), which is cleaved by a Ac-CoA acyltransferase (ACAA2, EC: 2.3.1.16) into propanoyl-CoA (**Prop-CoA**) and **Ac-CoA**. Similar to Leu metabolism, Ac-CoA can then be integrated into TCA cycle. **Prop-CoA** can be carboxylated by Prop-CoA carboxylase (PCCA, EC: 6.4.1.3) to form (S)-methylmalonyl-CoA (**(S)-MM-CoA**), which is then racemized by methylmalonyl-CoA epimerase (MCEE, EC: 5.1.99.1). The resulting (R)-methylmalonyl-CoA (**(R)-MM-CoA**) is then rearranged by methylmalonyl-CoA mutase (MUT, EC: 5.4.99.2) to form **Succ-CoA**, which can be utilized in the TCA cycle.

Val Metabolism:

**V2** is dehydrogenated by ACAD to form methacrylyl-CoA (**V3**). **V3** is then hydrated by HADHA to form (S)-3-hydroxyisobutyryl-CoA (**V4**). **V4** is consequently hydrolyzed by 3-hydroxyisobutyryl-CoA hydrolase (HIBCH, EC: 3.1.2.4). The released (S)-3-Hydroxyisobutyrate (**V5**) is dehydrogenated by 3-hydroxyisobutyrate dehydrogenase (HIBADH, EC:1.1.1.31) to form methylmalonate semialdehyde, which is further metabolized into methylmalonate by an aldehyde dehydrogenase (ALDH2, EC:1.2.1.3), but it can also be used to generate **Prop-CoA** by methylmalonate-semialdehyde dehydrogenase (ALDH6A1, EC: 1.2.1.27), which follows the same pathway as in Ile metabolism. From methylmalonate, methylmalonyl-CoA synthetase (ACSF3, EC: 6.2.1.-) forms **(R)-MM-CoA**, which is similarly to the Ile metabolism, rearranged to **Succ-CoA** and integrated into the TCA cycle.

Ketogenic and glucogenic behavior of BCAAs:

Ac-CoA and acetoacetyl-CoA are precursors for the synthesis of ketone bodies. Ketone bodies are an alternative energy source for the body, especially the brain, when glucose availability is low, such as during prolonged fasting or in certain metabolic conditions like a ketogenic diet. Based on the formation of these molecules in the catabolism of Leu and Ile, the two BCAAs are considered ketogenic (Zhang et al. 2018b).

Succinyl-CoA is a precursor for gluconeogenesis. The conversion to glucose can be useful when the body is low on glucose and needs the energy elsewhere. Since both Val and Ile produce succinyl-CoA, both BCAAs are considered glucogenic (Zhang et al. 2018b).

#### Whole-body fate of BCAAs

The whole-body fate of BCAAs was extensively reviewed by Blair et al. (Blair et al. 2021). Briefly, they proposed three key factors contributing to the flux of BCAA oxidation: (1) the tissue-specificity of BCAA oxidation in comparison to other energy sources, such as Gln and glucose, (2) overall mitochondrial oxidation and (3) the total tissue mass.

Supporting data of murine models with stable isotope labeling suggest that BCAA oxidation is generally rather low relative to other energy sources and the fractional contributions are highly tissue-specific (Neinast et al. 2019; Hui et al. 2020). The specific BCAA oxidation flux (measured in nmol/min/g) was determined to be the highest in the heart and brown adipose tissue (BAT) followed by the pancreas and kidney. Considering the total weight of the organs, skeletal muscles contribute by far the most to BCAA oxidation followed by BAT and the liver with approx. 59%, 19% and 8%, respectively.

Besides mitochondrial oxidation, Neinast et al. determined the whole-body fate of BCAAs with respect to integration into proteins using their murine model. The tissue-specific integration of BCAA was the highest in the pancreas, which aligns with the highest fractional rate of protein synthesis observed across all tissues. The pancreas also contributes predominantly to total protein integration (24%) considering the weight of the organ, next to the liver (27%) and muscle (24%). Overall, no correlation was identified between tissue-specific BCAA oxidation and protein integration across the different tissues tested (Neinast et al. 2019).

#### Sensing—mTOR

**Fig. 4:**
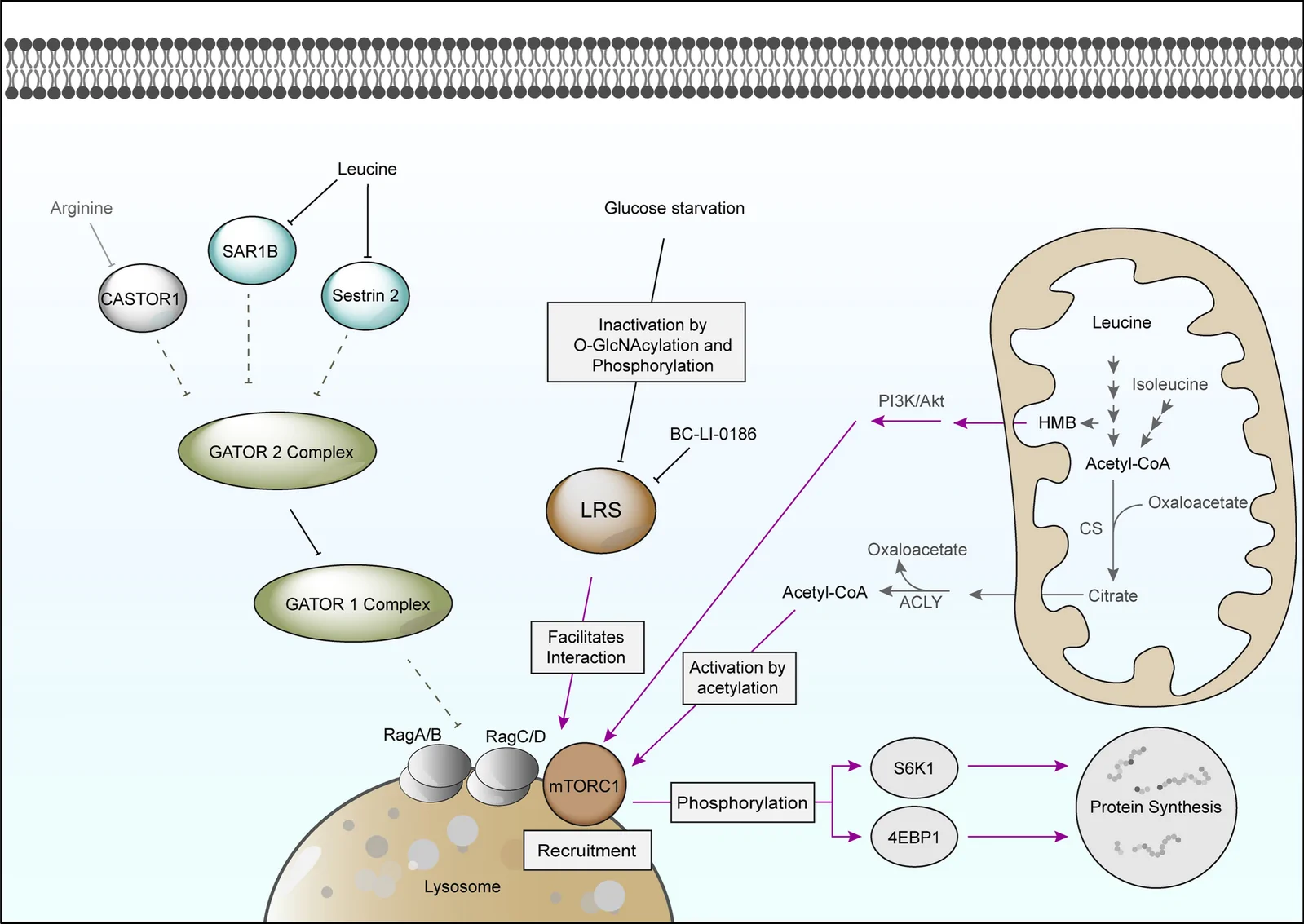
Activation of mTORC1 by Leu through GATOR complexes and LRS as well as Leu metabolites, namely HMB and acetyl-CoA. Red arrows indicate activation. Dotted lines represent inhibitory actions during Leu starvation, whereas solid lines indicate inhibition in case high Leu concentrations. 4EBP1, eukaryotic initiation 4E-binding protein 1; ACLY, ATP citrate lyase; Akt, protein kinase B; CASTOR1, cytosolic arginine sensor for mTORC1 subunit 1; CS, citrate synthase; GATOR, GTPase-activating protein towards Rags; HMB, β-hydroxy-β-methylbutyrate; LRS, leucyl-tRNA synthetase 1; mTORC1, mammalian target of rapamycin; PI3K, phosphatidylinositol-3 kinase; SAR1B, secretion associated Ras related GTPase 1B; S6K1, ribosomal protein S6 kinase 1

Leu plays a central role in promoting protein synthesis via the activation of the mTOR signaling pathway (Fig. 4) with two different kinase complexes, mTORC1 and mTORC2, each having distinct roles and substrate specificities. mTOR is a key master growth regulator able to sense the environment. In response to growth factors, cellular stresses, nutrient and energy level inputs, mTOR potentiates either anabolic processes, such as mRNA translation or lipid synthesis, or inhibit catabolic processes, such as autophagy or degradation through the proteasome. As a result, at low nutrient levels, including AAs, inactivation of mTOR leads to increased proteolysis and thereby intracellular AA levels are replenished, while high nutrient levels lead to activation of mTOR resulting in inactivation of autophagy and enhanced protein synthesis, e.g. for muscle growth. The following discussion will primarily focus on the connections with Leu, and for a broader understanding, it is recommended to consult other comprehensive reviews (Anthony et al. 2000, 2001; Kimball and Jefferson 2002; Saxton and Sabatini 2017; Wolfson and Sabatini 2017; Liu and Sabatini 2020; Takahara et al. 2020; Vellai 2021; Yue et al. 2022; Goul et al. 2023).

mTORC1 is sensitive to rapamycin and promotes protein synthesis via phosphorylating key regulators such as ribosomal protein S6 kinase 1 (S6K1) and eukaryotic initiation factor 4E-binding protein 1 (4E-BP1), which target downstream substrates associated with ribosome biogenesis, initiation and elongation factors as well as the mRNA splicing machinery. mTORC2 is insensitive to rapamycin and is involved in the regulation of the actin cytoskeleton and cell survival. It also plays a role in insulin signaling by phosphorylating Akt (Protein Kinase B) (Dodd and Tee 2012).

The Rag GTPase family, comprising RagA, RagB, RagC and RagD, are the basis to form the docking site on the lysosome for mTORC1. RagA or RagB pairs with either RagC or RagD and the nucleotide-loading state of the Rag GTPases determines mTORC1’s localization and activity (Sancak et al. 2010). When AAs (especially Leu and arginine) are abundant, RagA and/or RagB are bound to GTP, while RagC and/or RagD are bound to GDP. In this configuration, the Rag heterodimer can recruit mTORC1 to the lysosomal surface, where mTORC1 is activated by the small GTPase Rheb (Ras homolog enriched in brain) (Bar-Peled et al. 2013). Once mTORC1 is activated, it initiates mRNA translation and promotes protein synthesis via phosphorylating its downstream targets, S6K1, and 4E-BP1. In conditions of AA deprivation, the nucleotide-loading state is reversed: RagA and/or RagB binds GDP, while RagC and/or RagD binds GTP. This configuration prevents mTORC1 recruitment to the lysosome, thereby keeping mTORC1 inactive. The two stages are interconverted as a result of inter-subunit communication modulated by a number of regulating processes, including Leu-related sensing pathways (Shen et al. 2017).

Recent research suggests two pathways of mTOR modulation by Leu (Kume et al. 2010; Durán et al. 2012; Taylor 2014), the positive regulation of mTORC1 through leucyl-tRNA synthetase 1 (LRS) (Lee et al. 2018) and the negative regulation through inhibition by Sestrin2 (Chantranupong et al. 2014; Saxton et al. 2016; Wolfson et al. 2016) and SAR1B (Chen et al. 2021).

LRS is a cytosolic member of the class I aminoacyl-tRNA synthetase family and catalyzes the ATP-dependent attachment of Leu to its cognate tRNA. In search of the Leu recognition site, which mediates mTOR activation, LRS was initially ruled out by Lynch et al. due to discrepancies of LRS activity and mTOR activation through Leu analogues accompanied by limited alternative hypotheses at the time (Lynch et al. 2000). Their hypothesis relied on the synthetase activity of LRS (specifically tRNA charging) as initiator of Leu-sensed signaling. However, LRS was demonstrated to control the Leu-dependent mTORC1 pathway activation through direct binding to Rag GTPase and facilitated GTP hydrolysis of RagD (Han et al. 2012; Choi et al. 2017). Despite identification of the binding site to Rag and knowledge about the structural changes upon binding of Leu to LRS, the exact mechanism by which the enzyme facilitates conversion of RagD-GTP to RagD-GDP is not fully understood (Han et al. 2012; Kim et al. 2021; Raevsky et al. 2023). This sensing pathway is thought to be specific for Leu/LRS as isoleucyl-tRNA synthetase does not interact with Rag (Lee et al. 2018). Kim et al. showed that the small molecule BC-LI-0186, is capable of inhibiting the LRS/Rag interaction and consequently decreasing mTORC1 activity, but not the catalytic activity of the leucyl-tRNA synthetase (Kim et al. 2017). Recently, Raevsky et al. used *in silico* methods to simulate structure and functionality of LRS and screened for further putative inhibitors to potentially disrupt the interaction of LRS with mTOR, however, the molecular structures themselves were not explicitly mentioned (Raevsky et al. 2023).

In addition to its Leu sensing capabilities, LRS was reported to affect mTORC1 activity based on the glucose availability. Under glucose starvation, O-GlcNAcylation by OGT1 occurs on LRS at S1042, which results in local changes in the tertiary structure, impacting its ability to bind to RagD. This change in LRS O-glycosylation leads to the inactivation of mTORC1 (Kim et al. 2022), while the leucylation activity is maintained. Unc-51 Like Autophagy Activating Kinase 1 (ULK1), a protein kinase activated through the AMPK pathway (Kim et al. 2011) by low glucose levels and resulting in autophagy stimulation, phosphorylates LRS at S720. This site is crucial for Leu binding, and consequently its phosphorylation diminishes Leu sensing (Yoon et al. 2020). Kim et al. have shown that O-GlcNAcylation of LRS is a regulator of its own phosphorylation at S720, as it modulates the interaction with ULK1 (Kim et al. 2022). Altogether, this example highlights the interdependency between Leu and glucose sensing pathways, leading to mTOR regulation.

Sestrin2 functions as a second intracellular Leu sensor that negatively regulates the mTORC1 signaling pathway through the GATOR complexes. GATOR2 consists of 5 proteins and acts upstream of GATOR1, which inhibits the formation of heterodimers of RagA and RagB. While Sestrin2 was reported to interact with SEH1L, one of the components of GATOR2, the strength of this interaction was demonstrated to be dependent on the AA levels. Thus elevated, intracellular concentrations of Leu block inhibitory effects of Sestrin2 on GATOR2 complex, which helps in activating mTORC1. Similar effects have been observed upon binding of SAR1B (a small GTPase involved in vesicle formation) on MIOS, another component of GATOR 2. Under conditions of Leu deficiency, SAR1B inhibits mTORC1 by physically targeting GATOR2. In conditions of Leu sufficiency, SAR1B binds to Leu, undergoes a conformational change and dissociates from GATOR2, which results in mTORC1 activation (Chantranupong et al. 2014; Saxton et al. 2016; Wolfson et al. 2016; Chen et al. 2021). Notably, Leu is not the only AA impacting mTOR signaling. Similarly to Sestrin2, CASTOR1, a GTPase activating protein, was reported to interact with the GATOR2 complex. This interaction was disrupted in presence of high arginine concentrations, thus establishing CASTOR 1 as an arginine sensor for the mTORC1 pathway (Bar-Peled et al. 2013; Lee et al. 2018).

Finally, some products of the BCAA metabolism were reported to participate in mTOR modulation. For instance Ac-CoA, a Leu metabolite, inhibits autophagy through a positive modulation of mTORC1 activity (Mariño et al. 2014; Son et al. 2020), indicating the existence of a direct link between Leu metabolism and mTOR, that bypasses intermediary sensing mechanisms. More specifically, Ac-CoA stimulates the acetyltransferase EP300, which acetylates Raptor, a subunit of mTORC1. Raptor thereby increases its interaction with the Rag complex compared to an unacetylated Raptor, leading to mTORC1 tethering to the lysosomes and activation. However, the Ac-CoA mediated acetylation of Raptor was found to be cell type dependent (Son et al. 2019) and thus a careful verification of the mechanisms regulating autophagy under nutrient restricted conditions is needed for new cell lines. Potentially, this dependency can be traced back to different contributions of Leu to Ac-CoA production in different tissue and cell types. Notably, high amounts of supplemented Ile, but not Val, in AA starved conditions were also able to rescue mTORC1 activity (Son et al. 2019), which might be the result of Ac-CoA production from Ile metabolism.

Another metabolite of Leu, β-hydroxy-β-methylbutyrate (HMB), which is formed from **L3** by hydration, was suggested to influence mTORC1 activation in C2C12 myotubes. Administering 50 µM HMB resulted in higher Akt and mTOR activation. However, this effect was negated when LY294002, a PI3K inhibitor, was introduced, pointing to a PI3K/Akt-dependent mechanism for mTOR modulation (Kimura et al. 2014). This HMB influence on mTOR has been corroborated by other studies (Duan et al. 2018; Mu et al. 2023). While Ribeiro et al. demonstrated Akt and mTOR activation in rats following Leu administration, a direct connection with the Leu metabolite HMB was not established (Ribeiro et al. 2015). Suryawan et al. also observed mTOR activation due to HMB, yet couldn’t associate the mechanism with any recognized Leu-related routes (Suryawan et al. 2020).

### Involvement in synthesis of fatty acids

BCAAs have different connections to lipogenesis. Firstly, the Ac-CoA produced during the catabolism of Ile and Leu can be utilized to produce fatty acids (FA) (Crown et al. 2015) and cholesterol. Since these processes take place in the cytosol and the inner mitochondrial membrane is impermeable to Ac-CoA, Ac-CoA is condensed with oxaloacetate to form citrate by the enzyme citrate synthase. Citrate can then be transported across the mitochondrial membrane into the cytosol via the citrate transporter. Subsequently, citrate is cleaved by ATP-citrate lyase (ACLY) to regenerate oxaloacetate and Ac-CoA, which can then be used for even-numbered FAs (by fatty acid synthase (FASN)) and cholesterol synthesis (Kaneda 1991). In differentiated 3T3-L1 adipocytes, Crown et al. determined a fractional contribution of Leu, Ile and glutamine to lipogenic acetyl-CoA of 18%, 7% and 10% respectively, while the remaining acetyl-CoA was generated from glucose (Crown et al. 2015). Similar contributions of Ile and Leu were determined by Green et al. (Green et al. 2016).

The Prop-CoA formed from Ile and Val can also be utilized as first building block for FA synthesis, instead of being integrated into the TCA cycle (Crown et al. 2015). The propionyl-group is transported into the cytosol via the carnitine acetyltransferase (CrAT) (Violante et al. 2013) and generates odd-numbered, straight chain FAs. The investigations of Crown et al. have shown by MS fragmentation, that propionyl-CoA acts as primer and is sequentially elongated, and thus represents the carbons closest to the ω-end of the FA. Furthermore, Ile and Val were identified as unique precursors leading to the propionyl-CoA pool with approx. 67% and 33%, respectively (Crown et al. 2015).

Similarly, in adipocytes, the decarboxylation products formed by BCKDH, namely isovaleryl-CoA (**L2**), (S)-2-methylbutanoyl-CoA (**I2**) and isobutyryl-CoA (**V2**), can also be transported into the cytosol via CrAT (Violante et al. 2013) and can be used as initial building block for FA elongation by FASN. In these cases, the chain is branched and even-numbered with V2, but odd-numbered with I2 and L2. The resulting fatty acids can be classified as monomethyl branched-chain FAs (BCFA) and were found to be incorporated into lipid species, such as triacylglycerides and phospholipids. Synthesis of BCFAs was suggested to be tissue specific. De novo synthesis of BCFAs was shown by stable isotope labeling of Ac-CoA by supplementation of U-^13^C_6_-glucose, which was then incorporated in the synthesized FAs. In mice, this process was confirmed in white and brown adipose tissues, whereas the levels of BCFAs were rather low in brain and liver. This difference was attributed to lower expression of CrAT in brain and liver compared to adipose tissue. By administration of deuterated water, de novo synthesis of odd- and branched-chain fatty acids was also shown in human plasma in the range of 1–20 µM (Wallace et al. 2018). In cows, ruminant bacteria play a major role in production of BCFAs, which are then found in their milk. Vlaeminck et al. reviewed the effects of dietary treatment of cows on BCFAs in milk including further details on their synthesis (Vlaeminck et al. 2006).

A recent study elucidated a novel association between BCAA metabolism and de novo lipogenesis in rodent models for obesity, involving the regulation of BCKDH by BDK and PPM1K. Using phosphoproteomic analysis for liver samples, ACLY was demonstrated to be an alternate substrate for BDK and PPM1K (White et al. 2018). White et al. demonstrated that these regulatory enzymes are not confined to the mitochondria, where BCKDH is located, but are also present in the cytosol. Interestingly, unlike BCKDH, ACLY is activated by phosphorylation, indicating that BDK and PPM1K exert opposing effects on ACLY activity. ACLY plays a pivotal role in the production of cytosolic Ac-CoA, a fundamental precursor for the synthesis of FAs and cholesterol. In the rodent models, inhibition of BDK by BT2 or overexpression of PPM1K resulted in decreased circulating BCAA levels as well as reduced hepatic triglyceride levels (White et al. 2018). These findings underscore the multifaceted role of these regulatory enzymes in both BCAA metabolism and lipid synthesis.

### BCAAs in health and disease

As shown above, BCAAs are of high importance in human physiology due to their unique metabolic routes and multifaceted roles in protein synthesis, energy production, and signaling pathways. Disturbances in BCAA metabolism have been implicated in a range of pathological conditions, from insulin resistance and cancer to metabolic and neurodegenerative disorders. Furthermore, among other AAs, BCAAs have a significant impact on the gut microbiota and the immune system. BCAAs regulate immune cells and promote the expression of antimicrobial peptides (AMPs), which can influence the gut microenvironment and microbial composition. This interaction plays a critical role in modulating the gut-microbiome-immune axis under diverse physiological and nutritional conditions, contributing to intestinal homeostasis and overall health (Nie et al. 2018; Ma and Ma 2019). In the following, the role of BCAAs and their metabolism in the most prominent BCAA-related diseases as well as their use as food supplement will be displayed on a rather simplistic level. For more detailed insights, a range of recent reviews can be considered, a selection of which is shown in Table 2.

**Table 2:** Reviews including BCAA-related diseases

Publication title	Author, year of publication
The effect of branched-chain amino acid supplementation on cancer treatment	(Lee and Blanton 2023)
Branched-chain amino acids catabolism and cancer progression: focus on therapeutic interventions	(Xu et al. 2023)
Depiction of Branched-Chain Amino Acids (BCAAs) in diabetes with a focus on diabetic microvascular complications	(Tanase et al. 2023)
Amino acid catabolism: an overlooked area of metabolism	(Torres et al. 2023)
The role of branched chain amino acids metabolic disorders in tumorigenesis and progression	(Wang et al. 2022a)
Role of branched-chain amino acid metabolism in the pathogenesis of obesity and type 2 diabetes-related metabolic disturbances BCAA metabolism in type 2 diabetes	(Vanweert et al. 2022)
The role of branched-chain amino acids and branched-chain α-keto acid dehydrogenase kinase in metabolic disorders	(Du et al. 2022)
The mechanism of branched-chain amino acid transferases in different diseases: research progress and future prospects	(Nong et al. 2022)
The Critical Role of the Branched Chain Amino Acids (BCAAs) Catabolism-Regulating Enzymes, Branched-Chain Aminotransferase (BCAT) and Branched-Chain α-Keto Acid Dehydrogenase (BCKD), in human pathophysiology	(Dimou et al. 2022)
Prevention of loss of muscle mass and function in older adults during COVID-19 lockdown: potential role of dietary essential amino acids	(Park et al. 2022)
Leucine supplementation in cancer cachexia: mechanisms and a review of the pre-clinical literature	(Beaudry and Law 2022)
Oral branched-chain amino acids supplementation in athletes: a systematic review	(Martinho et al. 2022)
The effect of branched-chain amino acids supplementation in physical exercise: a systematic review of human randomized controlled trials	(Marcon and Zanella 2022)
Branched-chain amino acids and mitochondrial biogenesis: an overview and mechanistic summary	(Hinkle et al. 2022)
Branched-chain amino acids: catabolism in skeletal muscle and implications for muscle and whole-body metabolism	(Mann et al. 2021)
Insulin action, type 2 diabetes, and branched-chain amino acids: a two-way street	(White et al. 2021)
Emerging roles for branched-chain amino acid metabolism in cancer	(Sivanand and Vander Heiden 2020)
Multifaceted role of branched-chain amino acid metabolism in cancer	(Peng et al. 2020)
Regulation of skeletal muscle function by amino acids	(Kamei et al. 2020)
Leucine supplementation: a novel strategy for modulating lipid metabolism and energy homeostasis	(Zhang et al. 2020a)
Recent progress on branched-chain amino acids in obesity, diabetes, and beyond	(Siddik and Shin 2019)
Branched chain amino acids: beyond nutrition metabolism	(Nie et al. 2018)
Branched-chain amino acids in health and disease: metabolism, alterations in blood plasma, and as supplements	(Holeček 2018)
Branched-chain amino acids as critical switches in health and disease	(Zhang et al. 2018b)
Branched-chain amino acids and muscle protein synthesis in humans: myth or reality?	(Wolfe 2017)
Nutritional and regulatory roles of leucine in muscle growth and fat reduction	(Yin 2015)
Branched-chain amino acids in metabolic signaling and insulin resistance	(Lynch and Adams 2014)
Insulin resistance and the metabolism of branched-chain amino acids in humans	(Adeva et al. 2012)
Branched-chain amino acids: enzyme and substrate regulation	(Brosnan and Brosnan 2006)
Branched-chain amino acid metabolism: implications for establishing safe intakes	(Hutson et al. 2005)

Maple Syrup Urine Disease (MSUD) is a rare inherited metabolic disorder affecting 1 in 100,000 to 300,000 newborns (Piri-Moghadam et al. 2022a) and characterized by deficiencies in BCKDH (Yang et al. 2021; Billington et al. 2022; Yokoi et al. 2022). Besides mutations in BCKDH itself, mutations in PPM1K and BDK were found to cause mild MSUD (Oyarzabal et al. 2013; Maguolo et al. 2022; Ozcelik et al. 2023) and novel mutations are found quite frequently (Sun et al. 2020; Margutti et al. 2020; Medina et al. 2021; Fang et al. 2021; Li et al. 2023; Lashkarian and Salmani 2023). BCKDH deficiency leads to an accumulation of BCAAs incl. aIle (through keto-enol tautomerization and amination) and their keto acids in the blood and urine, giving a distinctive sweet odor reminiscent of maple syrup, from which the disease gets its name. In the healthy population, concentrations of Leu, Ile and Val are around 50–200 µM, 20–120 µM and 80–300 µM and aIle is only present at very low concentrations (usually < 2 µM) (Piri-Moghadam et al. 2022b). MSUD patients can have BCAA concentrations up to the sub-mM range (Kaur et al. 2020; Liu et al. 2022). Elevated levels of these substances, especially Leu and 2-oxoisocaproate, can be detrimental to the central nervous system and can lead to severe neurological complications. MSUD was reviewed in detail by multiple groups (Blackburn et al. 2017; Strauss et al. 2020; Xu et al. 2020; Amaral and Wajner 2022; Du et al. 2022; Rodrigues et al. 2022).

Type 2 diabetes mellitus (T2DM) is a multifactorial metabolic disorder characterized by chronic hyperglycemia resulting from impaired insulin activity and insulin secretion. The primary pathophysiological feature of T2DM is insulin resistance, where target tissues, especially liver, muscle, and adipose tissue, show reduced responsiveness to insulin. Obesity plays a significant role in the onset of insulin resistance. This diminished efficacy of insulin prompts the pancreas to produce more insulin, leading to hyperinsulinemia. Over time, pancreatic β-cells may become dysfunctional, reducing insulin secretion, which worsen the hyperglycemia. Consequently, obesity is a major risk factor and contributor to the development and progression of T2DM. Over the past few years, there have been studies that have shown a link between elevated BCAA levels and the risk of developing T2DM. However, it remains unclear, whether elevated BCAA levels are a cause or consequence of insulin resistance. Nevertheless, BCAAs can serve as markers of increased risk of insulin resistant T2DM (Bloomgarden 2018; White et al. 2021; Vanweert et al. 2022; De Bandt et al. 2022; Zhang et al. 2023; Supruniuk et al. 2023; Yao et al. 2023; Tanase et al. 2023).

BCAAs have been investigated in the field of oncology and were attributed a complex role in cancer development and progression. Altered BCAA metabolism has been linked to different aspects of tumor growth and metastasis. However, its role is still under discussion, as different research groups suggest opposing effects, either promoting or inhibiting tumor growth. For example, in breast cancer, elevated levels of BCAAs were shown to suppress both tumor growth and metastasis (Chi et al. 2022), while others attribute Leu’s mTORC1 activating property to increased protein synthesis and tumor progression, e.g. in colorectal cancer (Kang et al. 2024). In the latter case, impaired BCAA metabolism, in form of BCAT2 deficiency, was suggested to increase BCAA levels and activate mTORC1 leading to tumor growth. Notably, also BCAT1, BCKDH and its regulating enzymes were suspected to be involved in cancer promotion (Zhang and Han 2017; Ericksen et al. 2019; East et al. 2021; Wang et al. 2021b; Yang et al. 2022; Qian et al. 2023). Overall, the research is characterized by the dual nature originating from the opposing effects observed with BCAAs and their metabolism in cancer, which is addressed in more detail in a great number of reviews (selection shown in Table 2).

Due to the ability to activate mTOR and thereby to promote protein synthesis, BCAAs are favorable supplements stimulating muscle growth. This effect is favored by the comparably low catabolism of BCAAs in the liver, which allows for higher levels of these AAs in the muscle tissue. Furthermore, BCAAs can serve as energy source during exercise, reducing muscle fatigue and supporting endurance. Even though their effectiveness is still under debate, BCAAs have been popular in bodybuilding and athletic circles for several decades, but also to counteract muscle loss, e.g. as a result of natural aging, known as sarcopenia (Yin 2015; Moberg et al. 2016; Wolfe 2017; Jackman et al. 2017; Backx et al. 2018; Fuchs et al. 2019; Park et al. 2022; Marcon and Zanella 2022; Martinho et al. 2022; Caldo-Silva et al. 2023; Kaspy et al. 2023; Lee and Blanton 2023). While traditionally consumed as protein-rich foods, isolated forms, especially Leu, began to gain attention. As increased intake of an individual BCAA also results in increased catabolism and hence decreased plasma levels of the other two amino acids, which might then be unavailable for protein synthesis, it was recommended to at least supplement the three BCAAs in combination (Wolfe 2017), which should also hold true for the other essential AAs.

## Conclusion

The BCAAs, Leu, Ile and Val, are a distinct group of proteogenic AAs, that are essential to mammals and have shown high interdependency in their metabolism. They contribute to various cellular processes and were found to be involved in several pathological conditions.

In this review, we have summarized multiple aspects, which are relevant for different applications related to BCAAs. For most fields of research, rapid, but still reliable analytical methods are required. The isomerism of Leu, Ile and aIle is the major challenge, which can be overcome by state-of-the-art LC–MS approaches. Another obstacle caused by the structural similarity between the BCAAs is encountered during the industrial purification of crude material, that is produced from bacterial strains, such as *C. glutamicum*. However, besides cost-intensive chromatographic separation, complex crystallization processes have been developed to separate the poorly water-soluble BCAAs.

In physiology, the BCAAs have been of high interest in the past decades, not only as an important supplement in nutrition designed to boost muscle growth, but also in maintaining health and fighting disease. Here, we reviewed the uptake and distribution of BCAAs as well as their catabolism applicable to humans and other mammals. Ultimately, we summarized the status quo of research in various diseases. While the role of BCAAs and their disturbed catabolism in MSUD is very well understood and treatments are advanced and continuously improved, the contributions to other diseases, such as cancer, diabetes and obesity, are still contradictory and highly controversial. However, MSUD can undoubtedly be attributed to a dysfunction in a single enzyme complex, BCKDH, while the development of diabetes and the progression of different types of cancers are by far more complex.

Both in health and disease, an important role is attributed to the capability of cells to sense Leu and activate mTOR, a master regulator for protein synthesis and autophagy. Several connections between mTOR and Leu levels were established, most relevant of which are the proteins LRS as well as SAR1B and Sestrin2, where increased Leu levels ultimately lead to increased protein synthesis and vice versa. In the recent past, these mechanisms have been investigated and characterized further, e.g. by linking LRS with other processes, such as glucose uptake and BCAA-related metabolites. Furthermore, links to fatty acid synthesis were discovered, that could be relevant for understanding insulin resistance and obesity, such as the dual action of BDK and PPM1K on both BCKDH complex and ACLY. However, further understanding of these processes will be required in the future.

Ultimately, especially in medicine, it will be of utmost importance to further investigate and understand the role of BCAAs associated with pathological conditions to further improve health and well-being of people.

## Data Availability

No new data were created or analyzed in this study. Data sharing is not applicable to this article. No datasets were generated or analysed during the current study.

## Abbreviations

4EBP1: Eukaryotic initiation 4E-binding protein 1
AA: Amino acid
AA^0^: Neutral amino acid
AA^−^: Anionic amino acid
AA^+^: Cationic amino acid
ACAA2: Acetyl-CoA acyltransferase
ACAD: 2-Methylacyl-CoA dehydrogenase
ACAT: Acetyl-CoA C-acetyltransferase
Ac-CoA: Acetyl-coenzyme A
ACLY: ATP citrate lyase
ACSF3: Methylmalonyl-CoA synthetase
AHAS: Acetohydroxy acid synthase
aIle: Allo-Isoleucine
Akt: Proteine kinase B
ALDH2: Aldehyde dehydrogenase
ALDH6A1: Methylmalonate-semialdehyde dehydrogenase
AUH: Methylglutaconyl-CoA hydratase
BAT: Brown adipose tissue
BCAT: Branched-chain aminotransferase
BCAA: Branched-chain amino acid
BCFA: Branched-chain fatty acid
BCKA: Branched-chain keto acid
BCKDH: Branched-chain keto acid dehydrogenase
BDK: Branched-chain keto acid dehydrogenase kinase
CASTOR1: Cytosolic arginine sensor for mTORC1 subunit 1
CoA: Coenzyme A
CCS: Collision cross section
CrAT: Carnitine acetyltransferase
CS: Citrate synthase
DHAD: Dihydroxyacid dehydratase
ECD: Electron capture dissociation
ETD: Electron transfer dissociation
(r)ETF: (Reduced) electron-transfer flavoprotein
FA: Fatty acid
FAD: Flavin adenine dinucleotide
FASN: Fatty acid synthase
GABA: γ-Aminobutyric acid
GATOR: GTPase-activating protein towards Rags
GDH: Glutamate dehydrogenase
Gln: Glutamine
HADH: 3-Hydroxy-2-methylbutyryl-CoA dehydrogenase
HADHA: Enoyl-CoA hydratase
HIBADH: 3-Hydroxyisobutyrate dehydrogenase
HIBCH: 3-Hydroxyisobutyryl-CoA hydrolase
HILIC: Hydrophilic interaction chromatography
HMB: β-Hydroxy-β-methylbutyric acid
HMGCL: 3-Hydroxy-3-methylglutaryl-CoA lyase
Ile: Isoleucine
IPMDH: 3-Isopropylmalate dehydrogenase
IPMS: 2-Isopropylmalate synthase
IPMI: 2-Isopropylmalate isomerase
IVD: Isovaleryl-CoA dehydrogenase
KARI: Ketol-acid reductoisomerase
KB: 2-Ketobutyric acid
Leu: Leucine
LeuDH: Leucine dehydrogenase
LRS: Leucyl-tRNA synthetase 1
MCCC: β-Methylcrotonyl-CoA carboxylase
MCEE: Methylmalonyl-CoA epimerase
mTOR(C): Mammalian target of rapamycin (complex)
MS: Mass spectrometry
MS/MS: Tandem mass spectrometry
MSUD: Maple syrup urine disease
MUT: Methylmalonyl-CoA mutase
NAD^+^: Nicotinamide adenine dinucleotide (oxidized form)
NADPH: Nicotinamide adenine dinucleotide phosphate (reduced form)
OGDH: α-Ketoglutarate dehydrogenase
OXCT: 3-Oxoacid CoA-transferase
PCCA: Propionyl-CoA carboxylase
PDH: Pyruvate dehydrogenase
PI3K: Phosphatidylinositol-3 kinase
PLP: Pyridoxal phosphate
PPM1K: Protein phosphatase 1 K
Prop-CoA: Propionyl-coenzyme A
Pyr: Pyruvate
Rheb: Ras homolog enriched in brain
S6K1: Ribosomal protein S6 kinase 1
SAR1B: Secretion associated Ras related GTPase 1B
SLC: Solute carrier
Succ-CoA: Succinyl-coenzyme A
T2DM: Type 2 diabetes mellitus
TCA: Tricarboxylic acid
TD: Threonine dehydratase/deaminase
TDP: Thiamine diphosphate
TPP: Thiamine pyrophosphate
ULK1: Unc-51 like autophagy activating kinase 1
Val: Valine
